# Outer membrane vesicles generated by an exogenous bacteriophage lysin and protection against *Acinetobacter baumannii* infection

**DOI:** 10.1186/s12951-024-02553-x

**Published:** 2024-05-21

**Authors:** Changchang Li, Heng Xue, Xinjing Du, Raphael Nyaruaba, Hang Yang, Hongping Wei

**Affiliations:** 1 WHP Innovation Lab, National Key Laboratory of Special Pathogens and Biosafety, Wuhan Institute of Virology, Chinese Academy of Sciences, Wuhan, 430071 China; 2 University of Chinese Academy of Sciences, Beijing, 100049 China

**Keywords:** Outer membrane vesicles, Lysin, *Acinetobacter baumannii*, Immunization, Vaccine

## Abstract

**Background:**

The outer membrane vesicles (OMVs) produced by Gram-negative bacteria can modulate the immune system and have great potentials for bacterial vaccine development.

**Results:**

A highly active *Acinetobacter baumannii* phage lysin, LysP53, can stimulate the production of OMVs after interacting with *A. baumannii*, *Escherichia coli,* and *Salmonella*. The OMVs prepared by the lysin (LOMVs) from *A. baumannii* showed better homogeneity, higher protein yield, lower endotoxin content, and lower cytotoxicity compared to the naturally produced OMVs (nOMVs). The LOMVs contain a significantly higher number of cytoplasmic and cytoplasmic membrane proteins but a smaller number of periplasmic and extracellular proteins compared to nOMVs. Intramuscular immunization with either LOMVs or nOMVs three times provided robust protection against *A. baumannii* infections in both pneumonia and bacteremia mouse models. Intranasal immunization offered good protection in the pneumonia model but weaker protection (20–40%) in the bacteremia model. However, with a single immunization, LOMVs demonstrated better protection than the nOMVs in the pneumonia mouse model.

**Conclusions:**

The novel lysin approach provides a superior choice compared to current methods for OMV production, especially for vaccine development.

**Graphical Abstract:**

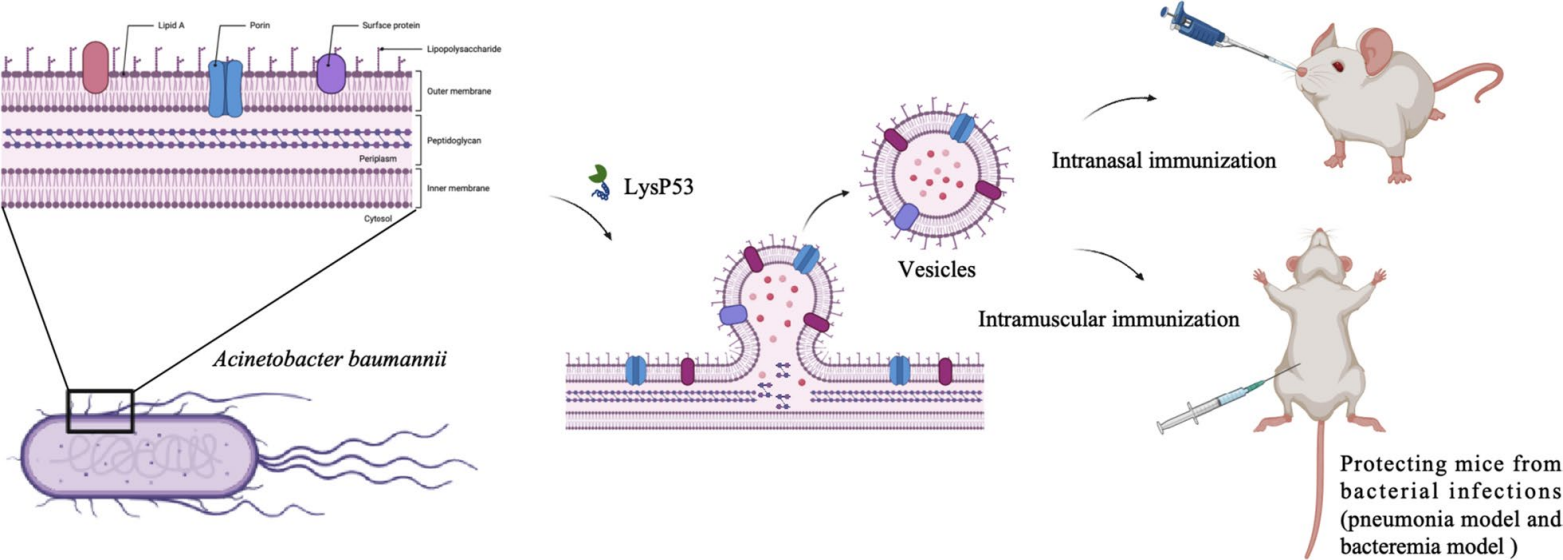

**Supplementary Information:**

The online version contains supplementary material available at 10.1186/s12951-024-02553-x.

## Introduction

Outer membrane vesicles (OMVs) are nanosized (20 nm to 250 nm) spherical proteoliposomes produced by Gram-negative bacteria during culture or infection [1]. Due to their composition of bacteria-derived antigens and multiple pathogen-associated molecular patterns (PAMPs) capable of modulating the immune system, OMVs have attracted interest in biotechnology applications, particularly in vaccine development [2–6]. For example, OMV vaccines targeting serogroup B strains of *Neisseria meningitidis* (MenB) have been licensed in Norway, Cuba, and New Zealand, leading to a substantial reduction in local cases of invasive meningococcal disease [7, 8].

Various methods exist for OMV production. Native outer membrane vesicles (nOMVs) or spontaneous OMVs (sOMVs) secreted naturally by Gram-negative bacteria for vaccine production are challenging to obtain and, if obtained, often yield low quantities [9–11]. Detergent extraction using sodium deoxycholate in the presence of EDTA, a widespread industrial method [12], has been used for decades to extract OMVs (dOMVs). However, the method has drawbacks, including the loss of negatively charged surface antigens [13, 14], aggregation and heterogeneity in size, resulting in the loss of OMVs during sterile filtration [14, 15], and contamination of OMV vaccines with cytoplasmic proteins due to bacterial cell lysis [16, 17]. To overcome these limitations, OMV‐generating bacteria are genetically engineered to improve OMV vaccine production [18–20]. However, this process is lengthy and unpredictable, requiring different modifications for different bacteria. And the resistance markers involved in genetic modification may be packaged during vesicle formation. Therefore, there needs to explore new ways to produce bacterial OMVs and study the changes in their composition and effects on immune efficacy.

Lysins are phage-encoded peptidoglycan hydrolases expressed in the late stages of phage infection. While their primary role is to lyse the host bacterial wall and release the daughter phage, recombinant lysins were found to also lyse the bacterial wall "from outside to inside", exhibiting efficient bactericidal activity and making it difficult for bacteria to develop resistance [21]. Lysins have attracted significant interests as potential novel antibacterial drugs, with several lysin drugs currently in phase 2 or 3 clinical trials [22]. These enzymes target peptidoglycan chain bonds in the bacterial cell wall and can be classified based on their catalytic domain activity, including n-acetyl-β-d-glucosaminidases, n-acetylmuramoyl-l-alanine amidases, N-acetylmuramidases, l-alanoyl-d-glutamate endopeptidases and interpeptide bridge-specific endopeptidases [23]. Both N-acetyl-β-d-glucosaminidases and n-acetylmuramidases act on the hemisaccharides in the bacterial cell wall, n-acetylmuramoyl-l-alanine amidases can hydrolyze the amide bond linking the sugar chain to the peptide group, and endopeptidases hydrolyze the amino or peptide linkages between the peptide side chains and the cross-linked bridges. Thus, phage lysins possess multiple enzymatic activity centers and can cleave different peptidoglycan chain bonds in the bacterial cell walls. Theoretically, OMVs containing different bacterial components may be obtained if different lysins were used to cut the peptidoglycan layer. However, no reports were found on utilizing lysin to produce OMVs.

In the current study, a highly active *Acinetobacter baumannii* phage lysin, LysP53, was found to stimulate the production of OMVs after interaction with *A. baumannii*, *Escherichia coli,* and *Salmonella*. Identified earlier in our laboratory, this lysin efficiently reduced bacterial counts from 10^6 ^CFU/mL to below 10 CFU/mL (below the detection limit) within 1 h [24]. Compared with naturally produced OMVs, lysin-OMVs exhibited significantly reduced endotoxin content and increased protein yield, suggesting that lysins may offer a new pathway for OMV production. Data from *A. baumannii* infection mouse models showed that intramuscular and intranasal immunization with lysin-OMVs provided equal or better protection than that of the native OMVs.

## Materials and methods

### Bacterial strains and culture conditions

All bacterial strains used in this study were cultured in lysogeny broth (LB) medium aerobically at 37 ℃. *A. baumannii* WHG40137, *E. coli* BL21 (DE3) and *S.* Enteritidis ATCC 13076 were used for purification of OMVs. *E. coli* BL21 (DE3) was used for protein expression. *A. baumannii* 3 and LB-6, and the *Staphylococcus aureus* ATCC 29213 were used in the binding experiments of antibody and bacteria.

### Preparation and purification of recombinant LysP53

*E. coli* BL21 (DE3) containing the plasmid pET28a-LysP53 was cultured to an OD600 of 0.5–0.6 in 500 mL of LB medium containing 50 μg/mL kanamycin and then induced with 0.5 mM isopropyl β-d-thiogalactoside at 16 ℃ for 16 h. Bacterial cells were then harvested by centrifugation at 7,370 × *g* for 10 min at 4 ℃, washed once with 20 mM imidazole, and then resuspended in 20 mM imidazole. The cells were lysed on ice by a cell disrupter and then centrifuged at 7370 × *g* for 20 min at 4℃. The resultant supernatant was filtered through a 0.22 μm syringe filter and subjected to a nickel nitrilotriacetic acid affinity column. LysP53 collected after eluting with 250 mM imidazole were pooled and dialyzed against 20 mM Tris-buffer (containing 0.5 mM NaCl, pH 6.8) overnight at 4 ℃.

### Preparation and purification of native OMVs (nOMVs)

To prepare native OMVs, a liter of LB medium was inoculated with 10 mL overnight cultures of *A. baumannii* WHG40137, *E. coli* BL21 (DE3), and *S.* Enteritidis ATCC 13076 before incubation at 37 ℃ overnight at 180 rpm. Resultant bacterial cells were pelleted by centrifugation at 10,000 × *g* for 10 min and the supernatants were filtered through a 0.22 μm membrane. The OMVs in the filtered solutions (1 L) was concentrated by ultrafiltration with 100 kD hyperfiltration tubes, washed with PBS three times, and subjected to ultracentrifugation at 200,000 × *g* for 90 min at 4 ℃ (Beckman, rotor TY90). The vesicle pellets obtained post centrifugation were resuspended in 1 mL PBS (pH 7.4) and the suspension was filtered through a 0.22 μm membrane. The absence of viable bacteria in the OMV preparations was determined by spreading aliquots on agar plates to test for bacterial growth.

### Preparation and purification of lysin stimulated OMVs (LOMVs)

To prepare LOMVs, overnight cultures of *A. baumannii* WHG40137, *E. coli* BL21 (DE3), and *S.* Enteritidis ATCC 13076 were pelleted by centrifugation at 10,000 × *g* for 10 min and the supernatant was removed. Then, a Tris–HCl solution (20 mM, pH 6.8) with 60 μg/mL LysP53 was used to resuspend the bacterial pellets. The bacterial suspensions were left to react with LysP53 for 2 h at room temperature, and then centrifuged at 10,000 × *g* for 10 min to collect the supernatant. After the supernatant was filtered through a 0.22 μm membrane, OMVs in the filtered solution was concentrated and washed with PBS buffer three times by ultrafiltration and subjected to ultracentrifugation at 200,000 × *g* for 90 min at 4 ℃ (Beckman, rotor TY90). The OMV pellets obtained post centrifugation were resuspended in 1 mL PBS (pH 7.4) and the suspension was filtered through a 0.22 μm membrane. The absence of viable bacteria in the OMV preparations was determined by spreading aliquots on agar plates to test for bacterial growth.

### Characterization of OMVs

The purified OMVs were characterized using several techniques summarized below to determine their morphology, size, protein (amount and composition), endotoxin level, and nucleic acid content. The morphology of the purified OMVs were visualized using a transmission electron microscope (Thermo Fisher Scientific, USA). Total protein of each OMV was used to estimate OMV yield, and the concentration of total protein was measured by a Pierce BCA protein assay (Thermo Fisher Scientific, USA) following the manufacturer’s instructions. Protein composition was determined by 12% SDS-PAGE stained with Coomassie blue. Endotoxin levels in OMV preparations were determined using a ToxinSensorTM Chromogenic LAL Endotoxin Assay Kit (Genscript, China) according to the manufacturer's instructions. The quantity of DNA in OMV preparations was determined using a Qubit 4 Fluorometer (Thermo Fisher Scientific, USA) according to the manufacturer’s instructions. Liquid chromatography mass spectrophotometry was performed by an external company (Wuhan GeneCreate Biological Engineering Co. Ltd) to determine individual proteins in the OMVs. The average particle size, polydispersity index and zeta potential value of OMVs were determined by dynamic light scattering using a Zetasizer Nano ZS90 machine (Malvern, USA). Rabbit anti His-Tag pAb (ABclonal, China) was used in western blot to verify if LOMV contain LysP53.

### Confirming OMV formation after lysin treatment with TEM

Briefly, *A. baumannii* bacterial cells were harvested after treatment with LysP53 and fixed with 2.5% glutaraldehyde for 1 h at room temperature. The fixed cells were further treated with 1% osmium tetroxide, dehydrated through a gradient series of ethanol concentrations (from 30 to 100%), and embedded with an Embed 812 kit (Electron Microscopy Sciences, Fort Washington, PA, USA). Ultrathin Sects. (60 to 80 nm) of the embedded specimens were prepared, deposited onto Formvar-coated copper grids (200 mesh), stained with 2% phosphotungstic acid (PTA, pH 6.8), and observed under a Tecnai TEM (FEI) operated at 200 kV.

### Generation of bone marrow dendritic cells (BMDCs) and cytotoxicity assays

To generate BMDCs, bone marrow cells isolated from the tibia and femurs of mice were cultured in RPMI 1640 medium containing 10% FBS, recombinant murine GM-CSF (6 ng/mL), and IL-4 (20 ng/mL) at 37 ℃, 5% CO_2_. The non-adherent cells were removed on day 5 and adherent cells were cultured in a fresh complete medium for another 2 days [25]. The generated BMDCs and commercial obtained RAW264.7 (∼1 × 10^4^ cells/well) were inoculated in a 96-well plate 1 day before the cytotoxicity experiment. The inoculated cells were then exposed to increasing concentrations of OMVs (0, 5, 10, 15, and 20 μg/mL) for 24 h, and residual cell viability finally determined by a Cell Counting Kit-8 (YEASEN, China) according to the manufacturer’s instructions. The relative viability of the cells after each treatment was normalized and compared to that of the PBS-treated control wells.

### FACS assay and cytokines assay of BMDCs

The fluorescence activated cell sorting (FACS) assay was performed on BMDCs to determine the activation of OMVs on antigen presenting cells. Briefly, BMDCs were both stimulated with either OMV (5 µg/mL) or PBS for 24 h. After three washes, BMDCs cells were incubated with FITC-CD11c, PE-CD40, PerCP/Cy5.5-CD80, and APC-CD86 before incubation in the dark for 30 min at 4 ℃. Flow cytometry data was acquired using a BD LSRFortessa instrument (BD Biosciences). To measure cytokines produced, BMDCs (2 × 10^6^ cells) were stimulated with either OMV (5 μg/mL) or PBS for 24 h. Concentrations of TNF-α, IL-12p70, IL-4, and IL-6 in the culture supernatants were detected by commercially obtained ELISA kits (ABclonal, China).

### Animal immunization

Two distinct groups of mice were subjected to different immunization protocols to assess the immune response. In the first group, the mice were immunized intramuscularly three times at intervals of 2 weeks with 10 μg OMVs absorbed to Imject Alum (Thermo Fisher Scientific, USA). In the second group, the mice were anesthetized with tribromoethanol and then intranasally immunized with 30 μL PBS containing 10 μg OMVs three times at intervals of 2 weeks. Following immunization, saliva, vaginal wash, and sera from the mice in both groups were collected for further analysis.

### Antibody production assay

Antibody levels after immunization were measured by standard indirect ELISA assay as previously described [26]. Briefly, 96-well flat-bottom microtiter plates were each coated with the OMV and incubated overnight at 4 ℃. Following blocking with 5% skim milk in PBST, the sera samples, pre-diluted at 1:100 were added to the microtiter plates. A subsequent 1:10 gradient dilution was done before incubation for 1 h at 37 ℃. HRP-conjugated goat anti-mouse IgG, IgG1, or IgG2a was used as a secondary antibody to determine antigen-specific IgG or IgG subtype level. Following incubation, 100 μL TMB substrate solution was added into each well and then kept in dark for 20 min. The reaction was finally quenched by a stop solution (2 M H_2_SO_4_). The optical density (OD) was measured at 450 nm using a microplate reader (Bio-Tek, USA). The sIgA level in the saliva and vaginal wash was measured following the instructions of an ELISA kit (FineTest, China).

### Western blots of proteins in OMVs

To analyze the presence of specific proteins in the purified OMVs, 0.5 μg of purified outer membrane proteins were separated on a 12% SDS-PAGE gel and transferred to a nitrocellulose membrane. Mouse sera collected 2 weeks after the last immunization (for both vaccinated and control mice) were used at a dilution of 1:1000 to bind with the proteins on the membrane. Finally, the bands were visualized through chemiluminescence after reaction with an HRP-labelled anti-mouse IgG antibody (Immunoway, USA).

### Binding between immunized mouse sera and *A. baumannii* cells

Cultured *A. baumannii* bacterial cells were washed with PBST through centrifugation three times. Subsequently, cells were incubated sequentially with immunized mouse sera, FITC-conjugated goat anti-mouse IgG, and DAPI for 1 h, 1 h, and 15 min, respectively. Finally, the samples were observed using a fluorescence confocal microscope (Nikon, Japan).

### Macrophage killing assay

Cultured *A. baumannii* bacterial cells were washed with PBS through centrifugation three times. Subsequently, they were added to a plate with RAW264.7 cells along with inactivated immunized mouse sera. After incubation for 5 h, the number of live bacteria was determined by plating serial dilutions on agar plates.

### Detection of splenocyte proliferation and cellular immune related factors

Two weeks following the last immunization, spleen cell suspensions of the immunized mice were prepared. The spleen cells (1 × 10^5^ cells/well) were stimulated with either OMV (5 μg/mL) or PBS for 48 h, respectively. The cell viability was assessed using a cell counting kit (Cell Counting Kit-8 (YEASEN, China)) following the manufacturer’s instructions. Simultaneously, concentrations of IFN-γ, IL-4, and IL-17A in the culture supernatants were determined using commercial ELISA kits (ABclonal, China).

### Mouse infection prevention and therapy

Two weeks following the last immunization, mice were anesthetized with an intraperitoneal injection of tribromoethanol and then challenged intranasally with either a sublethal dose of 2 × 10^9^ or an abdominally lethal dose of 2 × 10^8^ colony-forming unit (CFU) of *A. baumannii* WHG40137. The challenge doses were confirmed by CFU counts of serial tenfold dilutions on LB agar. The survival rates of the mice were monitored daily for 7 days. Additionally, bacterial burdens in the lungs and blood of mice with bacterial pneumonia were determined 24 h post the sublethal challenge.

To test the therapeutic effects of the immunized sera, BALB/c mice were anesthetized with tribromoethanol and intranasally challenged with a sublethal dose of 2 × 10^9^ CFU of *A. baumannii* WHG40137. After 24 h of the infection, 100 μL of either the immunized serum or the control serum was injected into each mouse via tail vein. The bacteria burden in the lungs of the mice was measured by plating serial dilutions on agar plates.

### Single-dose immunization experiment

BALB/c mice were anesthetized with tribromoethanol and intranasally immunized with 30 μL PBS containing 10 μg OMVs once. One week after immunization, mice were anesthetized with an intraperitoneal injection of tribromoethanol and then intranasally infected with 2 × 10^9^ CFU of *A. baumannii* WHG40137. Bacteria burden in the lungs of mice with bacterial pneumonia was determined 24 h post the sublethal challenge. Gene expressions were also measured using Bronchoalveolar lavage fluid (BALF) specimens from the singly immunized mice, as previously described [27]. The ^ΔΔ^Ct method was used to calculate the relative gene expressions using the primer sets in Table S1, targeting *TNF-α, IL-6, IL-1β, CYBB, S100A8,* and *mTOR* genes, with *β-actin* as the housekeep gene. The HiScript II One Step qRT-PCR SYBR Green Kit was used to determine the Ct values according to the manufacturer’s instructions (Vazyme, China).

### Statistical analysis

All the results were presented as the mean value plus a standard deviation (± S.D.) from at least three independent experiments. Statistical analyses were performed using the t-test. Values of *p < 0.05, **p < 0.01, ***p < 0.001 and ****P < 0.0001 were considered statistically significant.

## Results

### OMVs from different bacteria could be generated by LysP53 treatment

**Fig. 1:**
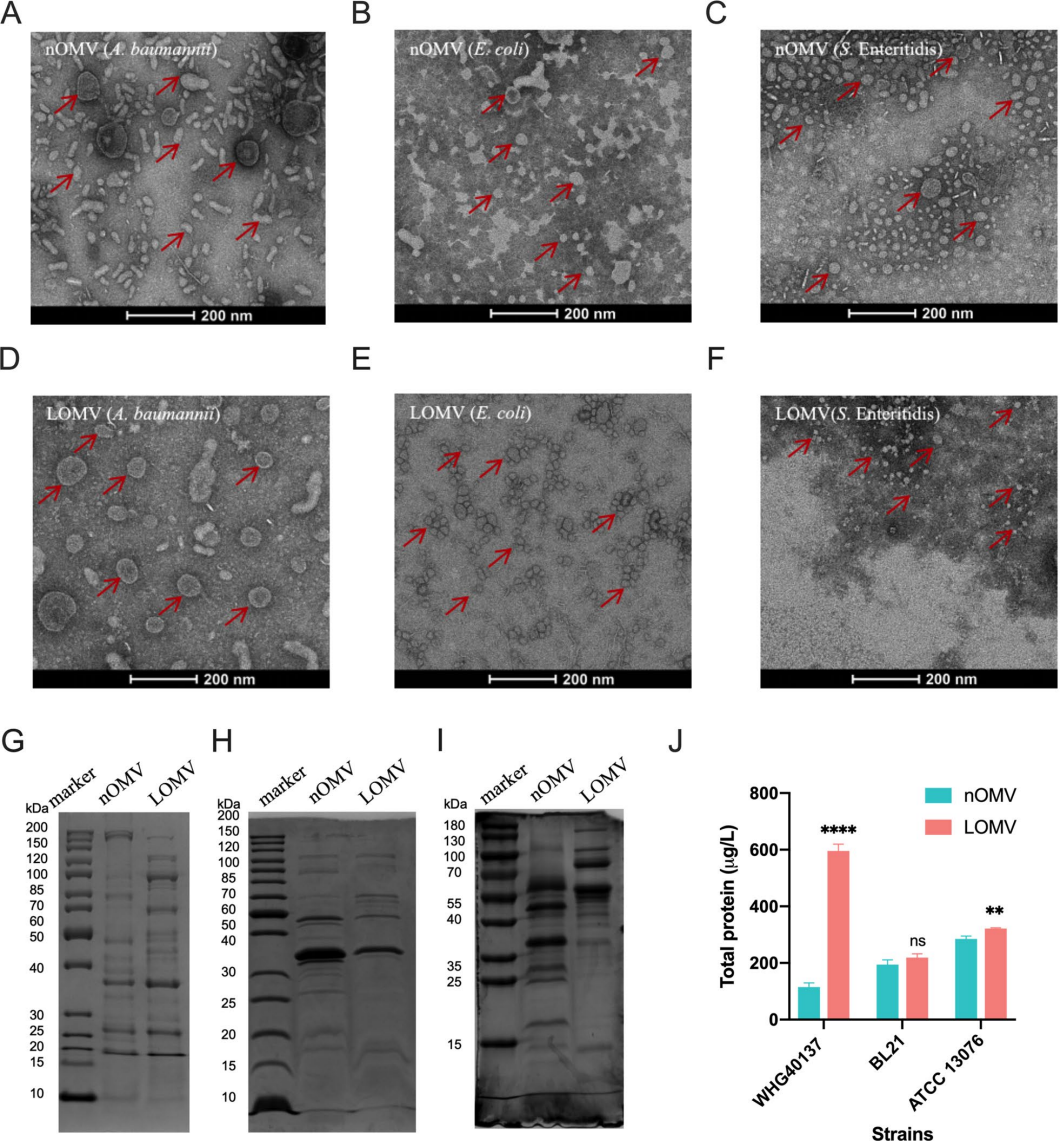
Outer membrane vesicles generated natively (nOMVs) and by LysP53 (LOMVs). **A**–**F** TEM images of nOMVs and LOMVs from *A. baumannii* WHG40137 (**A** and **D**), *E. coli* BL21 (DE3) (**B** and **E**), and *S.* Enteritidis ATCC 13076 (**C** and **F**); **G**–**I** SDS-PAGE analysis of protein compositions of nOMVs and LOMVs from *A. baumannii* WHG40137 (**G**), *E. coli* BL21 (DE3) (**H**) and *S.* Enteritidis ATCC 13076 (**I**); **J** Protein yields of nOMVs and LOMVs. **P < 0.01, ****P < 0.0001, and *ns* not significant

Three different Gram-negative bacteria *A. baumannii* WHG40137, *E. coli* BL21 (DE3) and *S.* Enteritidis ATCC 13076, were used to generate OMVs. Figures 1A–C illustrate the purification of native OMVs (nOMVs) from the culture supernatants of these three bacteria. Following treatment with LysP53, lysin-generated OMVs (LOMVs) from the same set of Gram-negative bacteria were obtained as shown in Figs. 1D–F. The generated LOMVs did not contain any LysP53 as seen in Figure S1. From the electron micrographs (Figs. 1A–F), both nOMV and LOMV had varied morphologies (rod- to spherical-like). Additionally, LOMVs were more homogenous in each bacterium compared to nOMVs. Notably, only LOMVs produced by *A. baumannii* WHG40137 were bigger in size compared to the nOMVs. The SDS-PAGE analysis of the two types of OMVs (Fig.  1G–I) showed distinct band patterns between LOMVs and nOMVs, indicating variations in protein compositions. It is noteworthy that the protein yields of the LOMVs were generally higher than those of the corresponding nOMVs **(**Fig. 1J), particularly evident for OMVs generated from *A. baumannii* WHG40137. The protein yield of LOMVs from *A. baumannii* WHG40137 was 595.54 ± 24.3 μg/L, more than five times that of nOMVs (115.25 ± 14.56 μg/L).

### Characterization of *A. baumannii* nOMV and LOMV

**Fig. 2:**
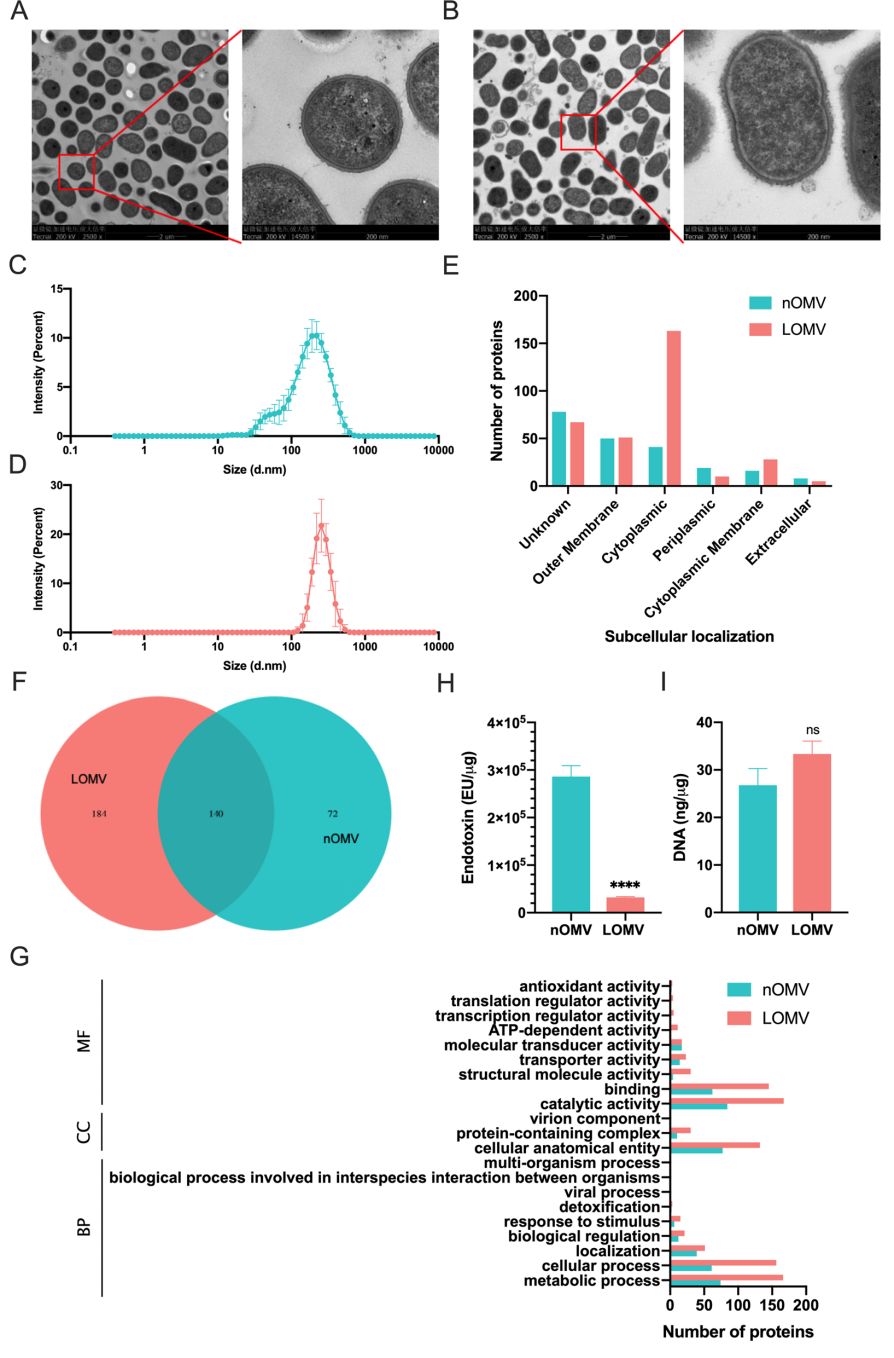
Characterization of nOMVs and LOMVs from *A. baumannii*. **A**, **B** The TEM images of bacterial cells producing nOMVs (**A**) and LOMVs (**B**); **C**, **D** Size distribution of nOMVs (**C**) and LOMVs (**D**) measured by dynamic light scattering; **E** Number of subcellular proteins detected by LC–MS; **F** Total number of proteins in both OMVs; **G** GO analysis of nOMV and LOMV. *BP* Biological process, *CC* cellular component, *MF* molecular function; **H** Endotoxin level of nOMVs and LOMVs; **I** DNA content of nOMVs and LOMVs. ****P < 0.0001, and ns, not significant

To further understand the characteristics of LOMVs compared to nOMVs, OMVs produced from *A. baumannii* WHG40137 were subjected to a detailed comparison. Under TEM, both nOMVs (Fig. 2A) and LOMVs (Fig. 2B) were observed as nano-sized outward bulges protruding from the surface of the *A. baumannii* cells. Dynamic light scattering analysis of the OMVs revealed that nOMV had an average diameter of 137.5 nm with a polydispersity index (PdI) of 0.303, while LOMV was larger, with an average diameter of 246.7 nm and a lower PdI of 0.089 (Figs. 2C and D). The lower PdI value of LOMV indicated a higher homogeneity compared to nOMV. nOMV and LOMV had the average zeta potential value of − 31.23 and − 36.63 mV, respectively. Further analysis of the proteins in OMVs using LC–MS showed that the LOMVs contain a significant higher number of cytoplasmic and cytoplasmic membrane proteins, but a smaller number of periplasmic and extracellular proteins than nOMVs (Fig. 2E). There were totally 212 proteins in nOMV and 324 proteins in LOMV, with 140 proteins present in both OMVs (Fig. 2F). Furthermore, a gene ontology (GO) analysis of the both proteins showed that LOMVs has a higher number of proteins contributing to biological processes, cellular components, and molecular function, compared to nOMV (Fig. 2G). LOMVs were also found to contain approximately 10 times lower endotoxin levels than nOMVs (Fig. 2H), with no significant difference in DNA content (Fig. 2I). In terms of cytotoxicity, LOMVs showed no cytotoxicity to both tested cells, while nOMV significantly affected cell activity with increasing concentration (Figure S2). To compare the maturation efficacy of dendritic cells by the two OMVs, BMDCs were stimulated with PBS, nOMV or LOMV for 24 h. FACS results revealed that a significant increased expression of costimulatory molecules (CD40, CD80, and CD86) was elicited by the two OMVs (Figures S3A–C). Additionally, treatment with OMVs resulted in the increased production of pro-inflammatory cytokine TNF-α. Significant secretion of Th1, Th2, and Th17 polarizing immune cytokines IL-12p70, IL-4, and IL-6 was also observed (Figures S3D–G), which suggested that OMVs had the potential to activate the innate immune response and T-cell mediated response.

### Protection of OMVs vaccination on mouse pneumonia model

**Fig. 3:**
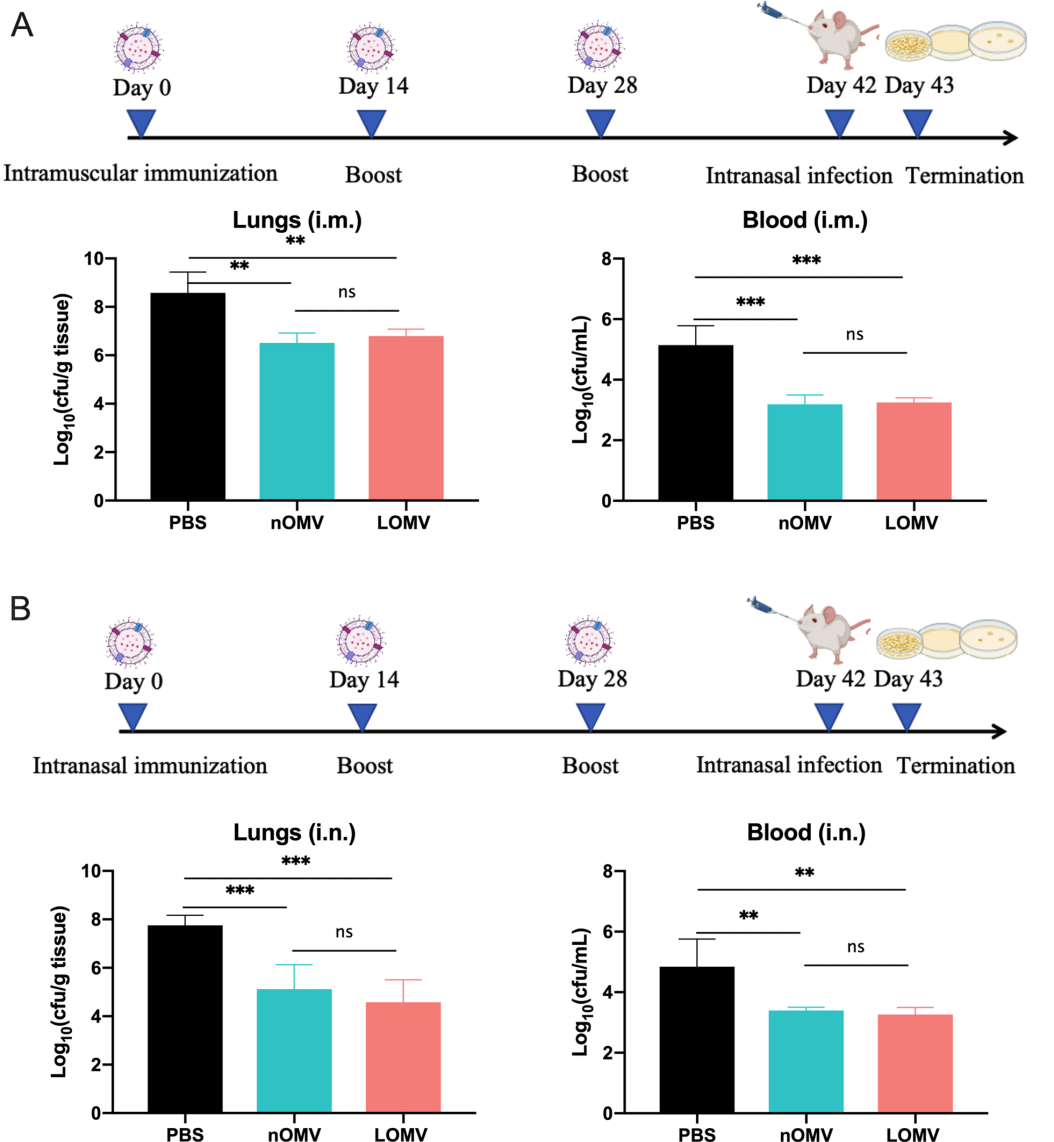
The protective effect of OMVs against a sub-lethal *A. baumannii* challenge. Bacterial loads in lungs and blood of the intramuscularly (**A**) and intranasally (**B**) immunized mice at 24 h post intranasal infection. Data are expressed as mean ± S.D., n = 5. **P < 0.01, ***P < 0.001, and ns, not significant

To evaluate the protective potential of the two OMVs (nOMVs and LOMVs), BALB/c mice were immunized either intramuscularly with the two OMVs absorbed to Alum adjuvant or intranasally with the two OMVs alone. A mouse pneumonia model was used to test the immuno-protection efficacy of OMVs after vaccination. Two weeks after the last vaccination, the mice were intranasally challenged with a sub-lethal dose of *A. baumannii* WHG40137. Bacterial burdens in the lungs and blood were determined 24 h after the challenge. As shown in Figs. 3A, B, vaccination with either type of OMVs resulted in over 90% clearance of *A. baumannii* WHG40137 from the lungs and the blood compared to the control. However, there was no significant difference between the nOMVs and the LOMVs vaccination groups.

### Protection of OMVs vaccination on mouse bacteremia model

**Fig. 4:**
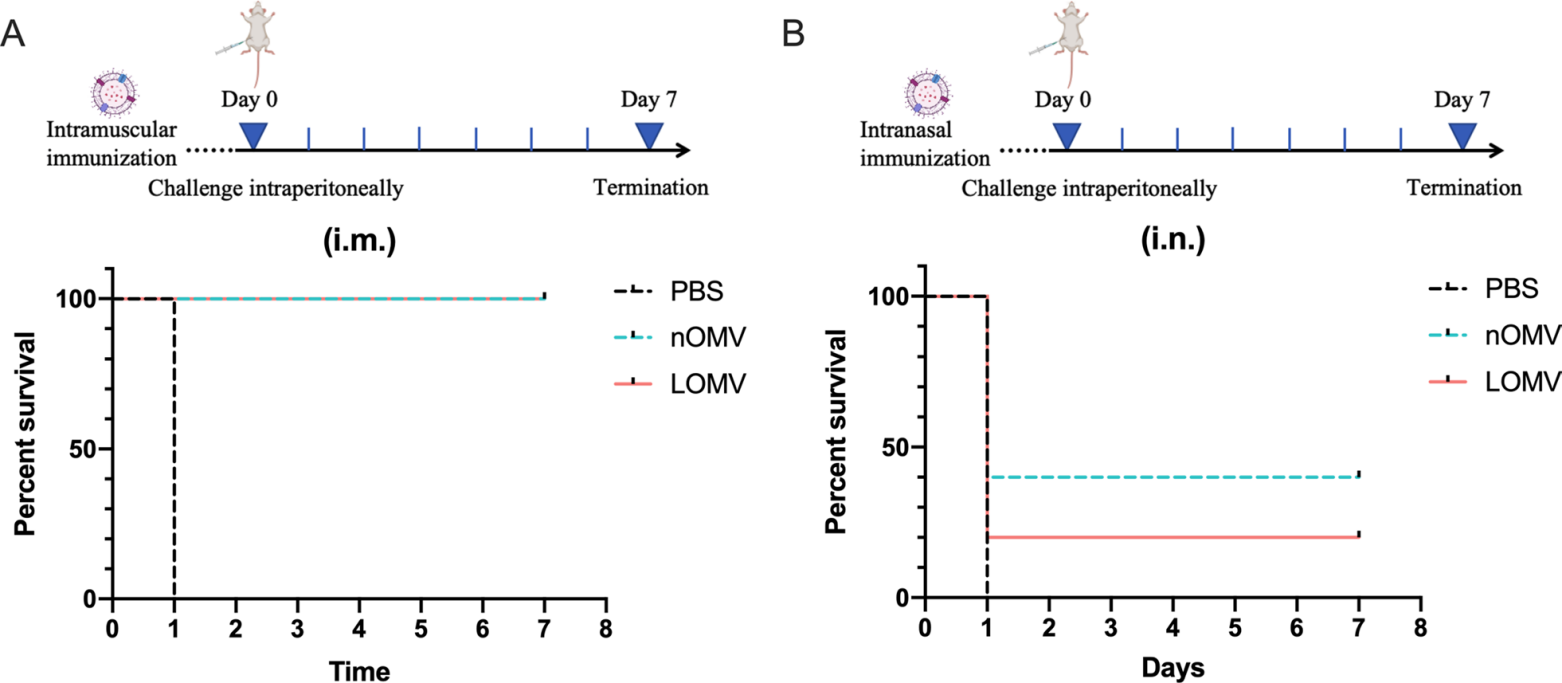
Survival rates of mice after OMVs vaccination against lethal *A. baumannii* challenge intraperitoneally. **A** Seven-days survival rates of the mice immunized intramuscularly; **B** Seven-days survival rates of the mice immunized intranasally. The number of mice in each group is 5

A mouse bacteremia model was also used to assess the immuno-protection efficacy of the OMVs (nOMV and LOMV) following intramuscular or intranasal vaccination. Two weeks after the last immunization, immunized and control mice were intraperitoneally challenged with a lethal dose of *A. baumannii* WHG40137, and the survival rates were monitored daily for 7 days post-challenge. Intramuscular vaccination with either nOMVs or LOMVs exhibited 100% protection of mice against the lethal challenge (Fig. 4A). In contrast, intranasal vaccination demonstrated lower protection rates, with only 40% for nOMV and 20% for LOMV (Fig. 4B).

### OMV vaccination via intramuscular route induced specific IgG and cellular immune responses

**Fig. 5:**
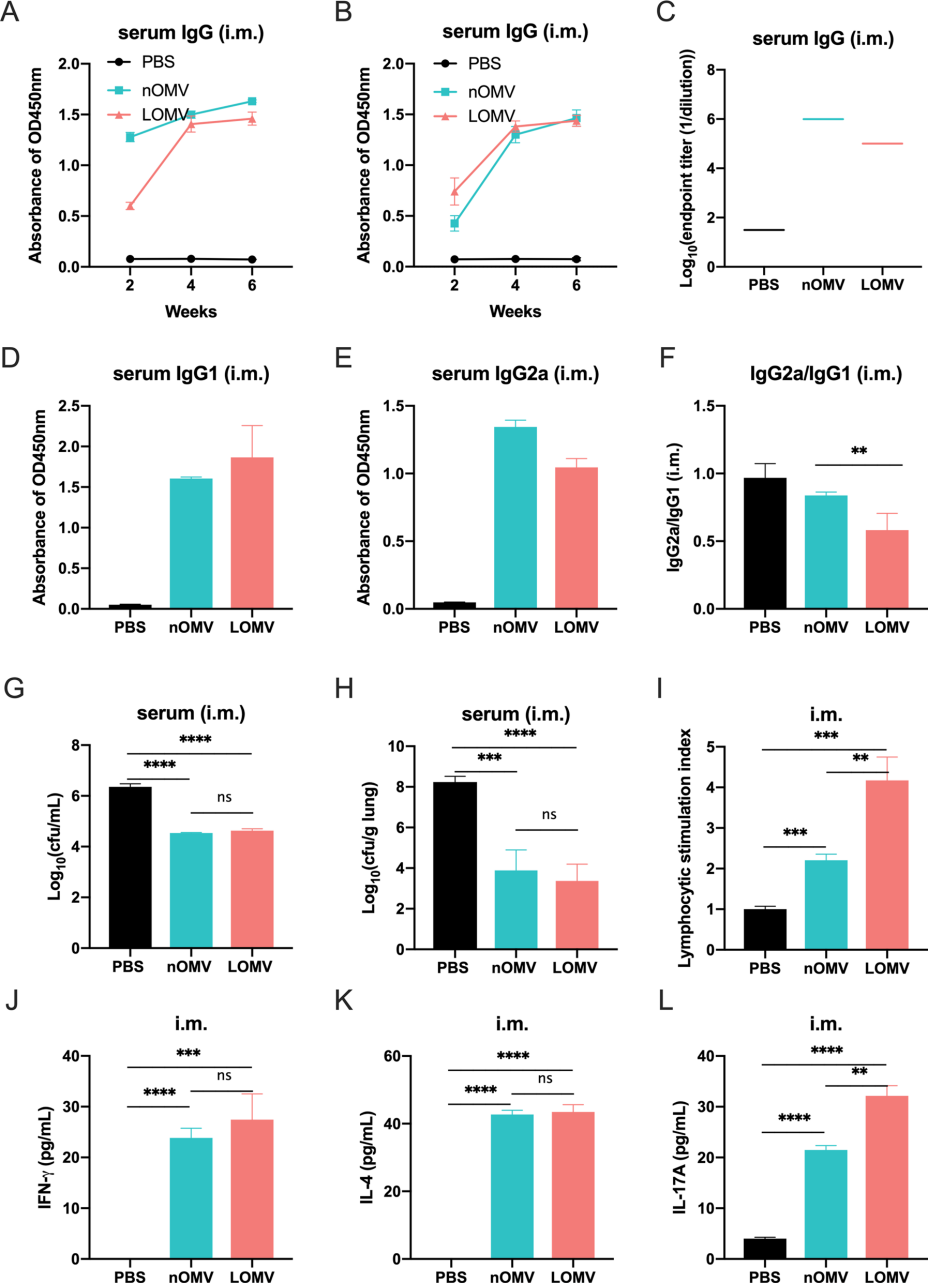
Humoral and cellular immune responses of mice intramuscularly immunized with nOMVs or LOMVs. **A** IgG antibody specific to nOMV during immunization; **B** IgG antibody specific to LOMV during immunization; **C** IgG antibody titers measured by ELISA after three times of immunization. The 96-well plate used for ELISA were coated with nOMV or LOMV, respectively, and the serum corresponds to those from mice immunized with nOMV or LOMV; **D**–**F** The levels of specific IgG1 (**D**) and IgG2a (**E**) in serum samples after immunization and the IgG2a/IgG1 ratio (**F**); **G** Lethal test of RAW264.7 macrophages; **H** Bacterial burden in the lungs of the infected mice treated with different sera; **I**–**L** Detection of splenic lymphocyte proliferation (**I**) and cellular immune related factors (**J**–**L**). Data are expressed as mean ± S.D., n = 5. **P < 0.01, ***P < 0.001, ****P < 0.0001, and *ns* not significant

To investigate the protective mechanism of OMVs against *A. baumannii* infection, the levels of specific serum IgG antibody in mice immunized intramuscularly were determined by ELISA. As depicted in Figs. 5A, B and S4, 2 weeks after the first (2 week) and the second (4 week) intramuscular immunization with OMVs, significant serum IgG antibody responses specific to both nOMVs and LOMVs were gradually elicited. The third injection did not further increase the IgG responses. The antibody titers produced by the nOMVs and LOMVs after three immunizations were 10^6^ and 10^5^, respectively (Fig. 5C). Markers for Th2 and Th1 responses, OMV-specific IgG1 and IgG2a levels were also determined (Figs. 5D and E). As shown in Fig. 5F, in all immunization groups, the IgG2a/IgG1 ratio was < 1, indicating a specific Th2-biased response when mice were immunized with both OMVs. Testing the functions of the antibodies in serum produced after intramuscular immunization with OMVs showed that the antibodies could bind with bacteria (Figure S6) and enhance the phagocytic function of the macrophages to kill *A. baumannii* (Fig. 5G). Moreover, in a mouse pneumonia model, as shown in Fig. 5H, the lung bacterial burden of mice treated with both OMVs serum was significantly lower than that of mice treated with PBS serum. Although the antibody levels induced by immunization with the LOMV were lower than those induced by nOMV, there was no significant difference in the therapeutic effect.

Under the stimulation of the corresponding antigens, the proliferation activity of the splenic lymphocytes isolated from the mice immunized with the LOMVs were much higher than that of those immunized with nOMVs, while both were significantly higher than that of the control mice immunized with PBS as shown in Fig. 5I. Immunization with nOMV and LOMV also stimulated spleen cells to secrete Th1, Th2, and Th17 polarizing immune cytokines IFN-γ, IL-4, and IL-17A as shown in Figs. 5J–L. It is worth noting that the secretion level of IL-17A from the cells in the LOMV group was significantly higher than that in the nOMV group (Fig. 5L).

### OMV vaccination via intranasal route induced specific IgG, sIgA and cellular immune responses

**Fig. 6:**
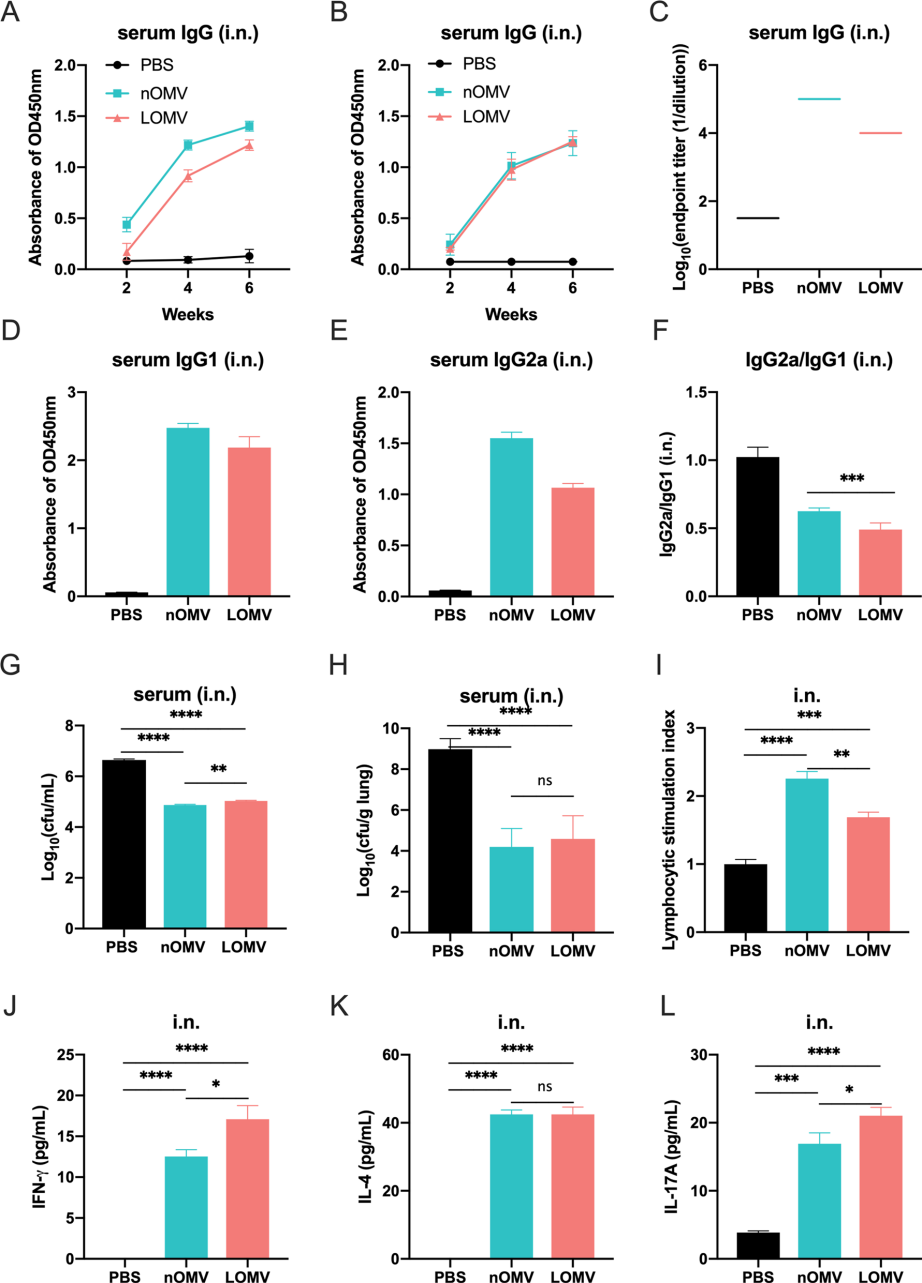
Humoral and cellular immune responses of mice intranasally immunized with either nOMV or LOMVs. **A** IgG antibody specific to nOMV during immunization; **B** IgG antibody specific to LOMV during immunization; **C** IgG antibody titers measured by ELISA after three times of immunization. The 96-well plate used for ELISA were coated with nOMV or LOMV, respectively, and the serum corresponds to those from mice immunized with nOMV or LOMV; **D**–**F** The levels of specific IgG1 (**D**) and IgG2a (**E**) in sera after immunization and the IgG2a/IgG1 ratio (**F**); **G** Lethal test of RAW264.7 macrophages; **H** Bacterial burden in the lungs of the infected mice treated with different sera; **I**–**L** Detection of splenic lymphocyte proliferation (**I**) and cellular immune related factors (**J**-**L**).Data are expressed as mean ± S.D., n = 5. *P < 0.05, **P < 0.01, ***P < 0.001, ****P < 0.0001, and *ns* not significant

Due to significant differences in the survival rate of mice with different immune routes in the mouse bacteremia model, the levels of specific serum IgG antibodies in the sera of the mice immunized intranasally were determined by ELISA. As shown in Figs. 6A, B and S5, intranasal immunization with either OMVs (nOMVs or LOMVs) elicited a significant serum-specific IgG antibody response compared to the PBS control group, with significant cross antibody reactions between the two OMVs. Unlike the intramuscular route, the third immunization further increased the IgG responses. The antibody titers produced by nOMV and LOMV after three immunizations were 10^5^ and 10^4^, respectively **(**Fig. 6C**)**, which are lower than those of intramuscular immunization (Fig. 5C). The levels of OMV-specific IgG1 and IgG2a were also determined (Figs. 6D and E), and similar to intramuscular immunization, specific Th2-biased responses were elicited when mice were intranasally immunized with both OMVs (Fig. 6F). We further tested the functions of the antibodies in the sera produced after intranasal immunization with the OMVs. The antibodies could bind with *A. baumannii* (Figure S7) and enhance the phagocytic functions of the macrophages to kill *A. baumannii* (Fig. 6G). Moreover, on the mouse pneumonia model, as shown in Fig. 6H, the lung bacterial burden of the mice treated with either OMVs serum was significantly lower than that of mice treated with the control PBS serum.

Testing the proliferation activity of the splenic lymphocytes isolated from immunized mice showed higher activities than that of the PBS control, as shown in Fig. 6I. However, in contrast to intramuscular immunization, the activities of the cells after immunization with nOMVs were significantly higher than those of the LOMVs. Intranasal immunization with the nOMVs and the LOMVs also stimulated spleen cells to secrete Th1, Th2, and Th17 polarizing immune cytokines IFN-γ, IL-4, and IL-17A as shown in Fig. 6J–L, with the secretion level of IL-17A from the cells in the LOMV group significantly higher than that in the nOMV group (Fig. 6L).

**Fig. 7:**
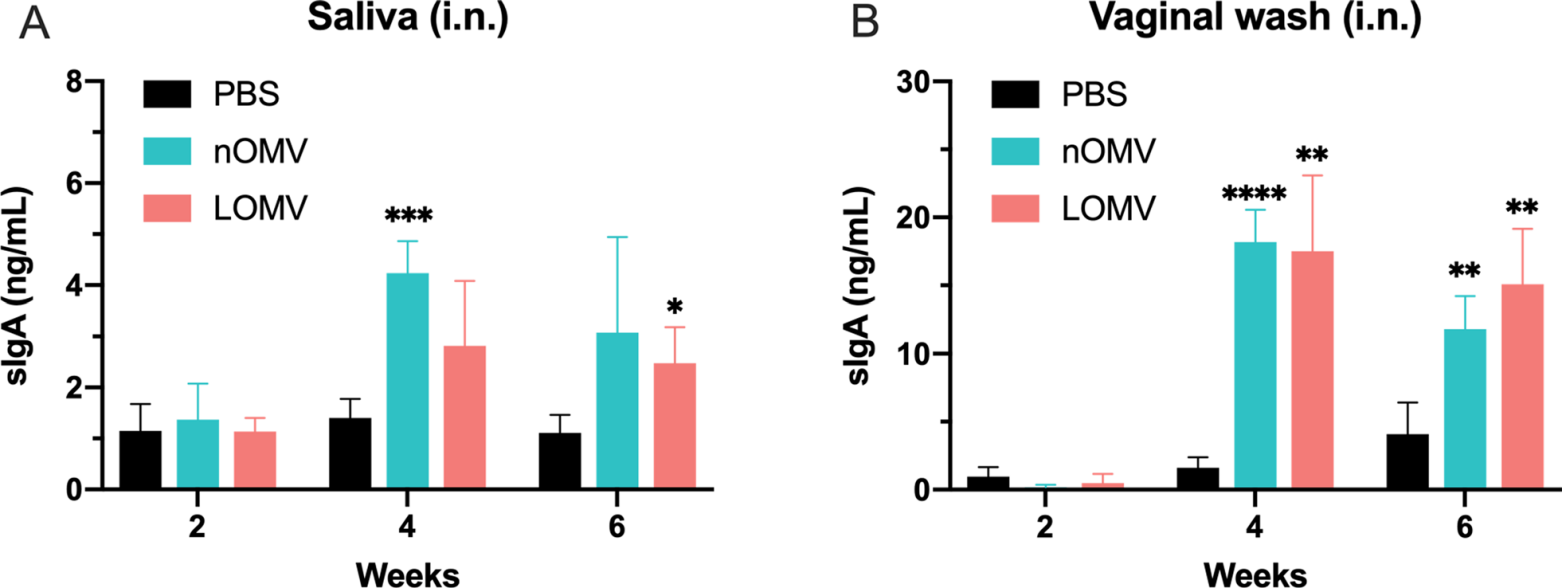
Specific sIgA immune responses elicited by intranasal immunization with either of the OMVs. BALB/c mice were immunized intranasally with OMVs. Saliva and vaginal wash were collected every 2 weeks. Secretory IgA levels in saliva (**A**) and vaginal wash (**B**) were measured by ELISA. Data are expressed as mean ± S.D., n = 4. *P < 0.05, **P < 0.01, ***P < 0.001, ****P < 0.0001, compared with PBS control

Considering that mucosal secretary IgA (sIgA) antibody played a role in blocking bacterial infection, secretions (saliva and vaginal wash) were collected to detect the level of sIgA after intranasal immunization. As shown in Figs. 7A and B, intranasal administration can induce sIgA antibody responses, which may be a possible reason why intranasal vaccination conferred protection in the mouse pneumonia model.

### Rapid protection from OMVs against *A. baumannii* pneumonia by a single intranasal immunization

**Fig. 8:**
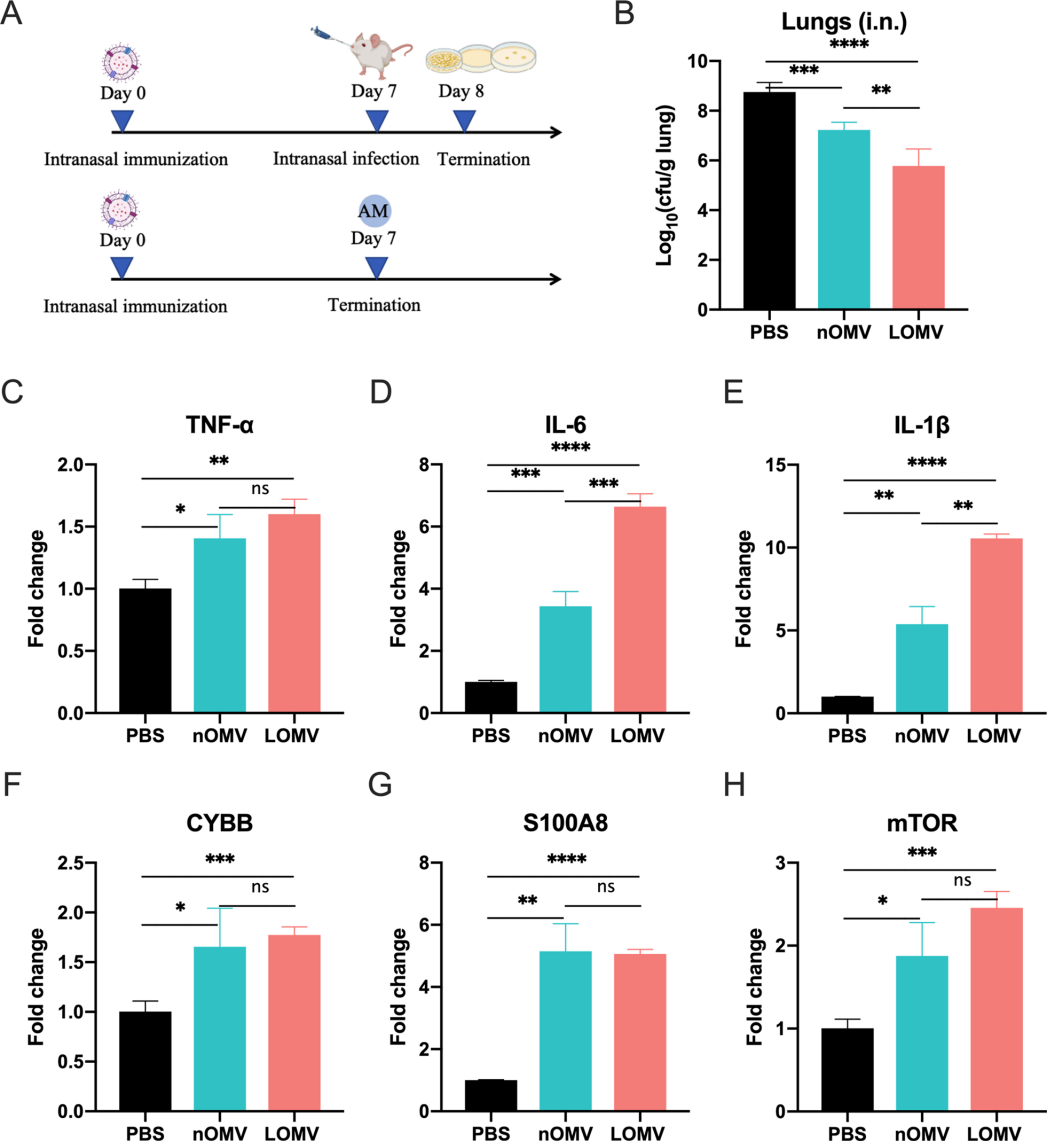
Protection against *A. baumannii* pneumonia by a single intranasal immunization with nOMV or LOMV. **A** Timeline representation of immunization and the experimental schedule; **B** Bacterial loads in the lungs of the immunized mice at 24 h post intranasal infection, n = 5; **C**–**H** Transcription level of cytokine genes in alveolar macrophages after 7 days of immunization, n = 10. *P < 0.05, **P < 0.01, ***P < 0.001, ****P < 0.0001, and *ns* not significant

To evaluate whether vaccination can elicit rapid protection, mice were intranasally immunized with the two OMVs (nOMV or LOMV) once and then infected intranasally with *A. baumannii* WHG40137 at the 7th day after the immunization (Fig. 8A). The bacterial load in the lungs of the mice immunized with the LOMVs was significantly lower than that of the nOMVs immunization, and both LOMVs and nOMVs groups had significantly lower bacterial loads than the PBS control group (Fig. 8B). Considering that alveolar macrophages (AMs) on the surface of the lung mucosa are the first line of defense against the invasion of pathogens, and more than 80% of cells in alveolar lavage fluid of normal mice are AMs, transcription levels of important cytokines of the AMs after 7 days of the immunization were measured (Figs. 8C–E). Compared with the PBS group, either nOMV or LOMV immunization could lead to increased transcription of *TNF-α*, *IL-6* and *IL-1β*, while LOMVs immunization could induce even higher transcription of *IL-6* and *IL-1β* than nOMVs immunization. At the same time, the two OMVs immunization could significantly increase the transcription level of phagocyte membrane binding oxidase CYBB (Fig. 8F) and antibacterial peptide S100A8 (Fig. 8G), showing that the OMVs may play an immune-protective role by mediating memory AMs to enhance phagocytosis and bactericidal function. The transcription of *mTOR* was also enhanced after immunization, indicating that the *A. baumannii* OMVs may also play a role in the natural immune memory of AMs through the *mTOR* pathway (Fig. 8H).

## Discussion

Gram-negative bacteria have two membranes, the inner membrane (IM) and the outer membrane (OM) with a network of peptidoglycan (PG) and the periplasmic space in between. Small protrusions from the OM could pinch off and become extracellular vesicles called outer membrane vesicles (OMVs) [28]. Alterations to the PG structure can prevent lipoprotein attachment, reducing crosslinks between the IM and OM. This indirectly increases OMV formation due to OM instability [3]. Since applying the recombinant lysins externally can hydrolyze the PG layer of the cell membrane and kill bacteria, the interaction between lysins and bacteria might destabilize the membrane, leading to OMV generation. This study used LysP53, known for lysing Gram-negative bacteria [24], especially *A. baumannii*, to test this hypothesis and explore the possibility of lysin’s to generate OMVs for the first time.

As expected, the bacterial cells of *A. baumannii*, *E. coli*, and *Salmonella* can all be stimulated by LysP53 to produce LOMVs (Fig. 1), proving the universality of this approach. By observing the bacterial cells of *A. baumannii* before and after treatment with the lysin, we found that there was a significant increase in extracellular vesicles secreted by the bacterial cells, and no lysis of the bacterial cells was observed. The mass spectrometry results indicate that the LOMVs contain more cytoplasmic proteins. All these results indicate that the hypothesis may be true, i.e., lysins could interact with bacterial cells, affecting the cross-linking between the peptidoglycan and the outer membrane, thereby disrupting the stability of the cell membrane, resulting in more cytoplasmic proteins being encapsulated in the LOMV. More interesting, the LOMVs prepared by the lysin showed better homogeneity, higher protein yield, lower endotoxin content, and lower cytotoxicity compared to the nOMV. Because endotoxins are harmful to humans, the endotoxin content needs to be well controlled in bioproducts. The much lower endotoxin content in the LOMVs may be because LOMVs are produced after the culture liquid containing endotoxins secreted by *A. baumannii* during growth is removed as supernatant. All these features offer the lysin approach a better choice than current methods [29] to produce OMVs, especially for vaccine development.

In this study, the intramuscular immunization with either LOMVs or nOMVs for three times were found able to provide good protection against *A. baumannii* infections in both pneumonia and bacteremia mouse models, while the intranasal immunization could offer good protection in the pneumonia model, but weak protection in the bacteremia model (Figs. 3 and 4). These results showed that the LOMVs immunization could offer similar protection like the nOMVs, which has previously been demonstrated that intramuscular immunization could induce protective immunity in a mouse model of sepsis [30] and clearance of the *A. baumannii* challenge inoculum from the lung and the blood in the pneumonia model [29]. The protection could be due to that both OMVs could induce both humoral and cellular immune responses. A Th2-biased response was elicited by both OMVs immunization, characterized by higher levels of IgG1 and lower levels of IgG2a in the sera (IgG2a/IgG1 < 1), same to an earlier study by Lin et al. [31]. Cytokines play an important role in the activation, proliferation, differentiation, survival, and apoptosis of lymphocytes, affecting the normal immune response of the immune system. When the spleen cells isolated from the immunized mice were stimulated by OMVs, the up-regulated expression levels of IFN-γ, IL-4 and IL-17A indicated that Th1, Th2 and Th17 were all activated to inhibit the *A. baumannii* infection in the mice. Moreover, the immunized sera also showed certain effects for post-infection treatment of *A. baumannii*. It is worth noting that no matter through intramuscular or intranasal immunization routes, the level of IL-17A induced by the LOMVs was higher than that of the nOMV, which may be the partial reason why the LOMVs could provide protection like the nOMVs although the total IgG response of the LOMVs was lower than that of the nOMVs.

However, in the case of a single immunization, the LOMVs showed better protection than the nOMVs in the pneumonia mouse model (Fig. 8). This difference may be due to that they contain different proteins (Fig. 2E and F). The LOMVs contain significant higher number of cytoplasmic and cytoplasmic membrane proteins, but a smaller number of periplasmic and extracellular proteins than nOMVs. Therefore, the immune responses would be different due to different antigens in the OMVs. The higher transcription of *IL-6* and *IL-1β* after the LOMVs immunization than the nOMVs immunization (Fig. 8D and E) proved this. Hao et al. demonstrated that a single intranasal immunization of inactivated whole cell (IWC) induced rapid, efficient, and broad protection against certain Gram-negative bacterial pneumonia, which was dependent on trained innate immunity mediated by AMs [32]. We also found this phenomenon in the single immunization experiment of OMVs. It indicates that LOMVs may be a better choice to quickly stimulate the immune response of the body against *A. baumannii* infections and play a protective role when needed.

## Conclusions

In summary, this study introduces a novel approach using the highly active *Acinetobacter baumannii* phage lysin, LysP53, to stimulate OMV production. The lysin-induced OMVs offer equal or better protection than naturally produced OMVs in mouse models. More importantly, LOMVs showed better homogeneity, higher protein yield, lower endotoxin content, and lower cytotoxicity compared to nOMVs, making this approach a better choice than current methods to produce OMVs, especially for vaccine development. In future, we will explore applying this method and different lysins to Gram-positive bacteria to generate OMVs.

### Supplementary Information

Supplementary Material 1.

Supplementary Material 2. Identification and quantitative information of proteomics in nOMVs and LOMVs.

Supplementary Material 3. The predicted subcellular protein fractions of nOMVs.

Supplementary Material 4. The predicted subcellular protein fractions of LOMVs.

Supplementary Material 5. The GO analysis of nOMVs.

Supplementary Material 6. The GO analysis of LOMVs.

## Data Availability

All data generated or analysed during this study are included in this published article [and its supplementary information files].

## References

[1] Schwechheimer C, Kuehn MJ (2015). Outer-membrane vesicles from gram-negative bacteria: biogenesis and functions. Nat Rev Microbiol.

[2] Sartorio MG, Pardue EJ, Feldman MF, Haurat MF (2021). Bacterial outer membrane vesicles: from discovery to applications. Annu Rev Microbiol.

[3] Balhuizen MD, Veldhuizen EJA, Haagsman HP (2021). Outer membrane vesicle induction and isolation for vaccine development. Front Microbiol.

[4] Fang W, Jing Z, Li Y, Zhang Z, Lin Z, Yang Z, Tian Y, Zhang C, Ma Y, Hou L (2024). Harnessing enucleated cancer cells as trojan horse cell vaccines. Cell Rep Phys Sci.

[5] Lin Z, Meng F, Ma Y, Zhang C, Zhang Z, Yang Z, Li Y, Hou L, Xu Y, Liang X, Zhang X (2024). In situ immunomodulation of tumors with biosynthetic bacteria promote anti-tumor immunity. Bioact Mater.

[6] Meng F, Li L, Zhang Z, Lin Z, Zhang J, Song X, Xue T, Xing C, Liang X, Zhang X (2022). Biosynthetic neoantigen displayed on bacteria derived vesicles elicit systemic antitumour immunity. J Extracell Vesicles.

[7] Petousis-Harris H (2018). Impact of meningococcal group B OMV vaccines, beyond their brief. Hum Vaccin Immunother.

[8] Delbos V, Lemée L, Bénichou J, Berthelot G, Deghmane AE, Leroy JP, Houivet E, Hong E, Taha MK, Caron F (2013). Impact of MenBvac, an outer membrane vesicle (OMV) vaccine, on the meningococcal carriage. Vaccine.

[9] Gasperini G, Arato V, Pizza M, Aricò B, Leuzzi R (2017). Physiopathological roles of spontaneously released outer membrane vesicles of Bordetella pertussis. Future Microbiol.

[10] Gasperini G, Biagini M, Arato V, Gianfaldoni C, Vadi A, Norais N, Bensi G, Delany I, Pizza M, Aricò B, Leuzzi R (2018). Outer membrane vesicles (OMV)-based and proteomics-driven antigen selection identifies novel factors contributing to bordetella pertussis adhesion to epithelial cells. Mol Cell Proteom.

[11] Valguarnera E, Feldman MF (2017). Glycoengineered outer membrane vesicles as a platform for vaccine development. Methods Enzymol.

[12] Acevedo R, Fernández S, Zayas C, Acosta A, Sarmiento ME, Ferro VA, Rosenqvist E, Campa C, Cardoso D, Garcia L, Perez JL (2014). Bacterial outer membrane vesicles and vaccine applications. Front Immunol.

[13] van der Pol L, Stork M, van der Ley P (2015). Outer membrane vesicles as platform vaccine technology. Biotechnol J.

[14] van de Waterbeemd B, Streefland M, van der Ley P, Zomer B, van Dijken H, Martens D, Wijffels R, van der Pol L (2010). Improved OMV vaccine against Neisseria meningitidis using genetically engineered strains and a detergent-free purification process. Vaccine.

[15] van de Waterbeemd B, Zomer G, Kaaijk P, Ruiterkamp N, Wijffels RH, van den Dobbelsteen GP, van der Pol LA (2013). Improved production process for native outer membrane vesicle vaccine against Neisseria meningitidis. PLoS ONE.

[16] Klimentová J, Stulík J (2015). Methods of isolation and purification of outer membrane vesicles from gram-negative bacteria. Microbiol Res.

[17] van de Waterbeemd B, Mommen GP, Pennings JL, Eppink MH, Wijffels RH, van der Pol LA, de Jong AP (2013). Quantitative proteomics reveals distinct differences in the protein content of outer membrane vesicle vaccines. J Proteome Res.

[18] Pérez-Cruz C, Cañas MA, Giménez R, Badia J, Mercade E, Baldomà L, Aguilera L (2016). Membrane vesicles released by a hypervesiculating *Escherichia coli* Nissle 1917 tolR mutant are highly heterogeneous and show reduced capacity for epithelial cell interaction and entry. PLoS ONE.

[19] Schwechheimer C, Kuehn MJ (2013). Synthetic effect between envelope stress and lack of outer membrane vesicle production in *Escherichia coli*. J Bacteriol.

[20] Takaki K, Tahara YO, Nakamichi N, Hasegawa Y, Shintani M, Ohkuma M, Miyata M, Futamata H, Tashiro Y (2020). Multilamellar and multivesicular outer membrane vesicles produced by a buttiauxella agrestis tolb mutant. Appl Environ Microbiol.

[21] Gerstmans H, Criel B, Briers Y (2018). Synthetic biology of modular endolysins. Biotechnol Adv.

[22] Danis-Wlodarczyk KM, Wozniak DJ, Abedon ST (2021). Treating bacterial infections with bacteriophage-based enzybiotics: in vitro in vivo and clinical application. Antibiotics.

[23] Fenton M, Ross P, McAuliffe O, O'Mahony J, Coffey A (2010). Recombinant bacteriophage lysins as antibacterials. Bioeng Bugs.

[24] Li C, Jiang M, Khan FM, Zhao X, Wang G, Zhou W, Li J, Yu J, Li Y, Wei H, Yang H (2021). Intrinsic antimicrobial peptide facilitates a new broad-spectrum lysin LysP53 to kill acinetobacter baumannii in vitro and in a mouse burn infection model. ACS Infect Dis.

[25] Xue RY, Guo MF, Guo L, Liu C, Li S, Luo J, Nie L, Ji L, Ma CJ, Chen DQ (2019). Synthetic lipopeptide enhances protective immunity against helicobacter pylori infection. Front Immunol.

[26] Liu C, Luo J, Xue RY, Guo L, Nie L, Li S, Ji L, Ma CJ, Chen DQ, Miao K (2019). The mucosal adjuvant effect of plant polysaccharides for induction of protective immunity against Helicobacter pylori infection. Vaccine.

[27] Gu H, Liu D, Zeng X, Peng LS, Yuan Y, Chen ZF, Zou QM, Shi Y (2018). Aging exacerbates mortality of Acinetobacter baumannii pneumonia and reduces the efficacies of antibiotics and vaccine. Aging.

[28] Beveridge TJ (1999). Structures of gram-negative cell walls and their derived membrane vesicles. J Bacteriol.

[29] Li S, Chen DQ, Ji L, Sun S, Jin Z, Jin ZL, Sun HW, Zeng H, Zhang WJ, Lu DS (2020). Development of different methods for preparing Acinetobacter Baumannii outer membrane vesicles vaccine: impact of preparation method on protective efficacy. Front Immunol.

[30] McConnell MJ, Rumbo C, Bou G, Pachón J (2011). Outer membrane vesicles as an acellular vaccine against Acinetobacter baumannii. Vaccine.

[31] Lin L, Tan B, Pantapalangkoor P, Ho T, Hujer AM, Taracila MA, Bonomo RA, Spellberg B (2013). Acinetobacter baumannii rOmpA vaccine dose alters immune polarization and immunodominant epitopes. Vaccine.

[32] Gu H, Zeng X, Peng L, Xiang C, Zhou Y, Zhang X, Zhang J, Wang N, Guo G, Li Y (2021). Vaccination induces rapid protection against bacterial pneumonia via training alveolar macrophage in mice. Elife.

