# Supplementary material for: Outer membrane vesicles generated by an exogenous bacteriophage lysin and protection against Acinetobacter baumannii infection
Source: J Nanobiotechnology. 2024 May 21;22:273. doi: 10.1186/s12951-024-02553-x (PMC11110425; doi:10.1186/s12951-024-02553-x)
Supplement: Supplementary file 1 — Supplementary Material 1. [file 12951_2024_2553_MOESM1_ESM.docx]

**Supplementary Materials**

**Outer membrane vesicles generated by an exogenous bacteriophage lysin and protection against *Acinetobacter baumannii* infection**

Changchang Li^1,2^, Heng Xue^1,2^, Xinjing Du^1^, [Raphael Nyaruaba](https://www.ncbi.nlm.nih.gov/pubmed/?term=Nyaruaba%20R%5bAuthor%5d&cauthor=true&cauthor_uid=34917062)^1,2^, Hang Yang^1,2^, Hongping Wei^1,2*^

^1^WHP Innovation Lab, National Key Laboratory of Special Pathogens and Biosafety, Wuhan Institute of Virology, Chinese Academy of Sciences, Wuhan, 430071, China.

^2^University of Chinese Academy of Sciences, Beijing 100049, China.

*Correspondence: Hongping Wei, hpwei@wh.iov.cn; Tel.: +86-27-87998873

**Table S1 Primer sequences**

| Primer | Sequence, 5’~3’ |
| --- | --- |
| TNF-α-F  TNF-α-R  IL-6-F  IL-6-R  IL-1β-F  IL-1β-R  β-actin-F  β-actin-R  CYBB-F  CYBB-R  S100A8-F  S100A8-R  mTOR-F  mTOR-R | CCTATGTCTCAGCCTCTTCTCAT  CACTTGGTGGTTTGCTACGA  CCTCTCTGCAAGAGACTTCC  CTCCGGACTTGTGAAGTAGG  GGACCCCAAAAGATGAAGGGCTGC  GCTCTTGTTGATGTGCTGCTGCG  GGCTGTATTCCCCTCCATCG  CCAGTTGGTAACAATGCCATGT  CAAGTGCCCCAAGGTATCCAAGTT  TGAATAGCCCCTCCGTCCAGTCTC  TGCGATGGTGATAAAAGTGG  GGCCAGAAGCTCTGCTACTC  GTCTACTCGCTTCTATGACCAG  TTCCCACCTTCCACTCCTAT |


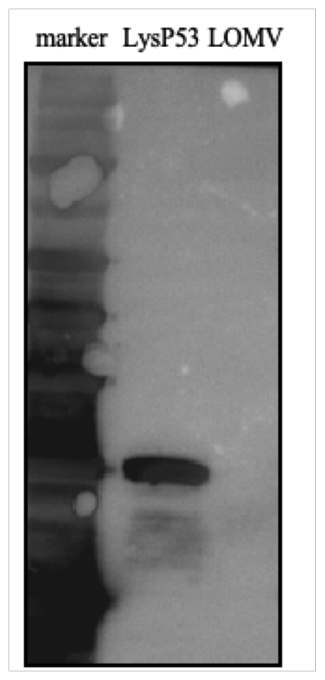


Figure S1. Western blot analysis confirming LOMV do not contain LysP53.


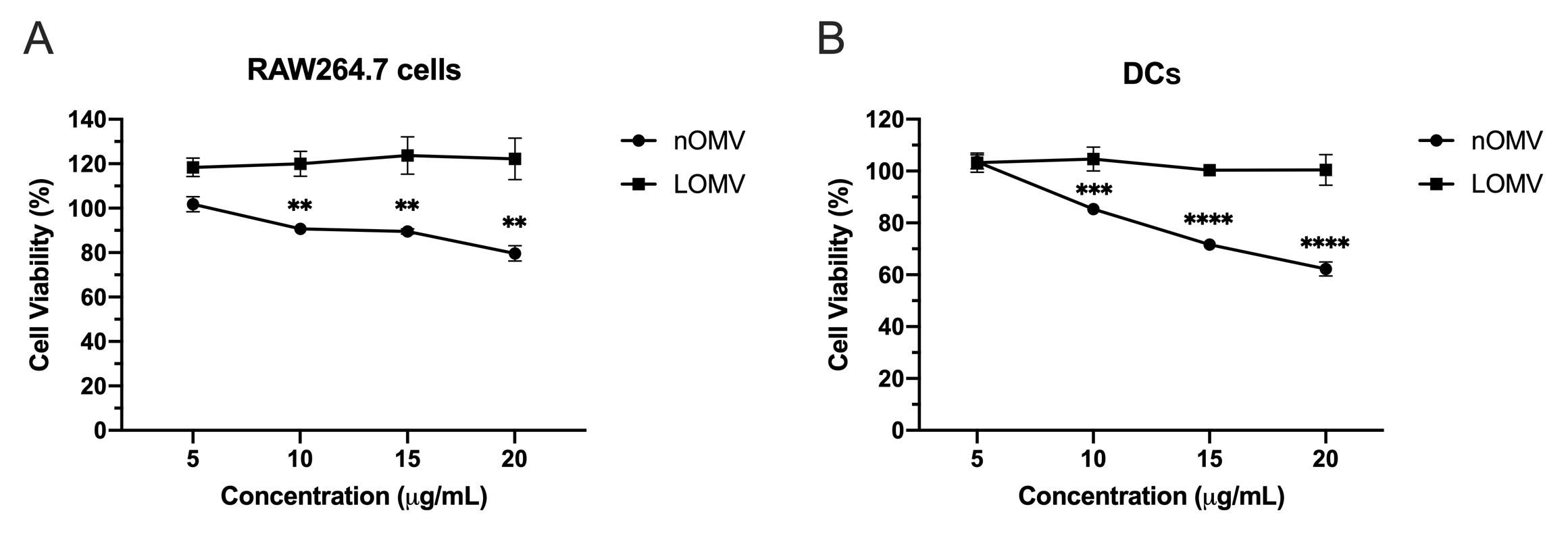


**Figure S2.** Cytotoxicity of OMVs. RAW264.7 cells (A) and DC cells (B) were co-cultured with different concentrations of OMVs for 24 h, residual cell viability was determined by Cell Counting Kit-8 assay.


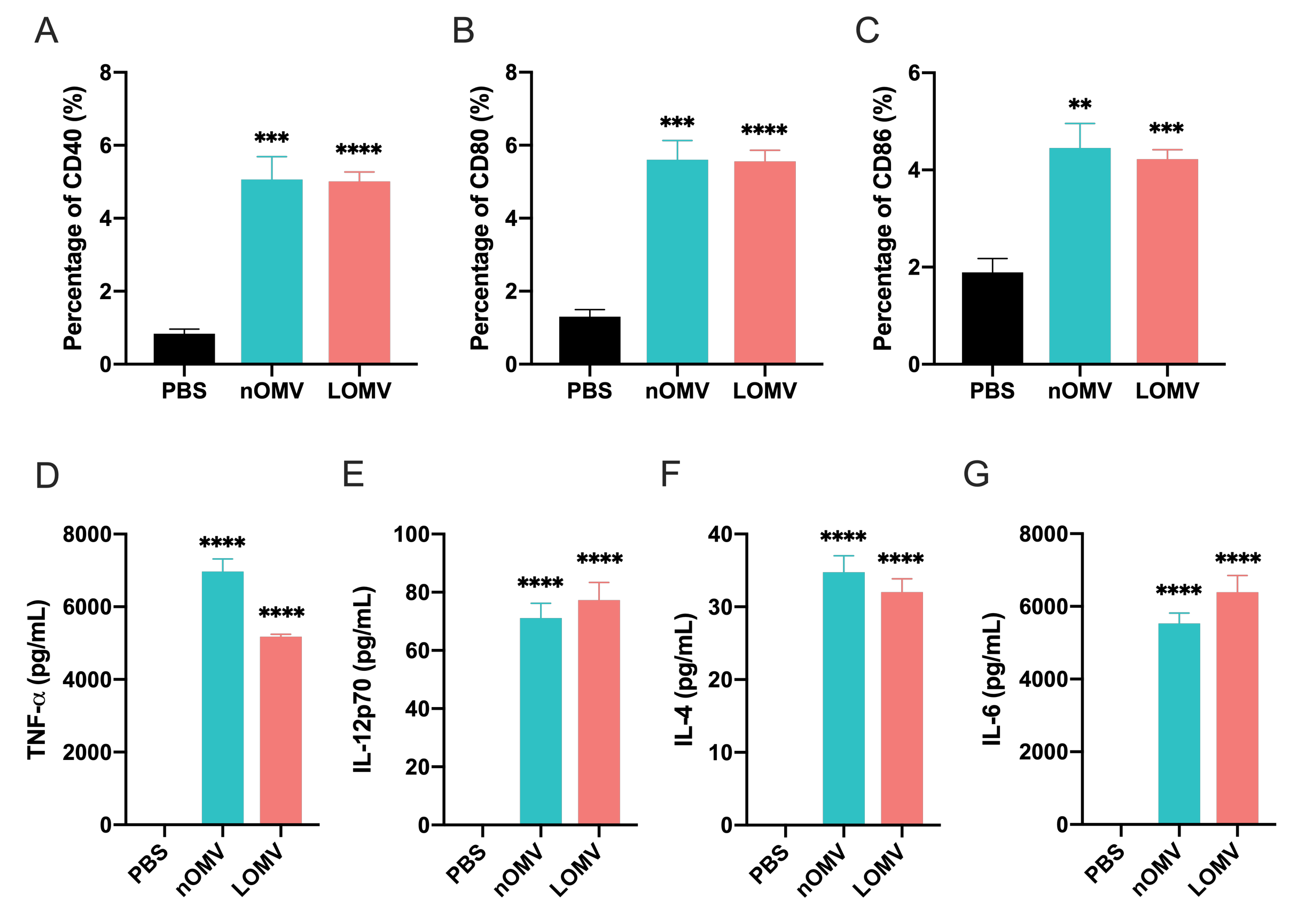


Figure S3. The expression of maturation surface markers in BMDCs treated with the OMVs and cytokines expression in the supernatants of BMDCs stimulated with the OMVs. (A–C) Normalized expression level of maturation markers. ELISA assays were used to measure the accumulation of TNF-α (D), IL-12p70 (E), IL-4 (F), and IL-6 (G) in the supernatants of culture. **P < 0.01, ***P < 0.001, ****P < 0.0001, compared with PBS control.


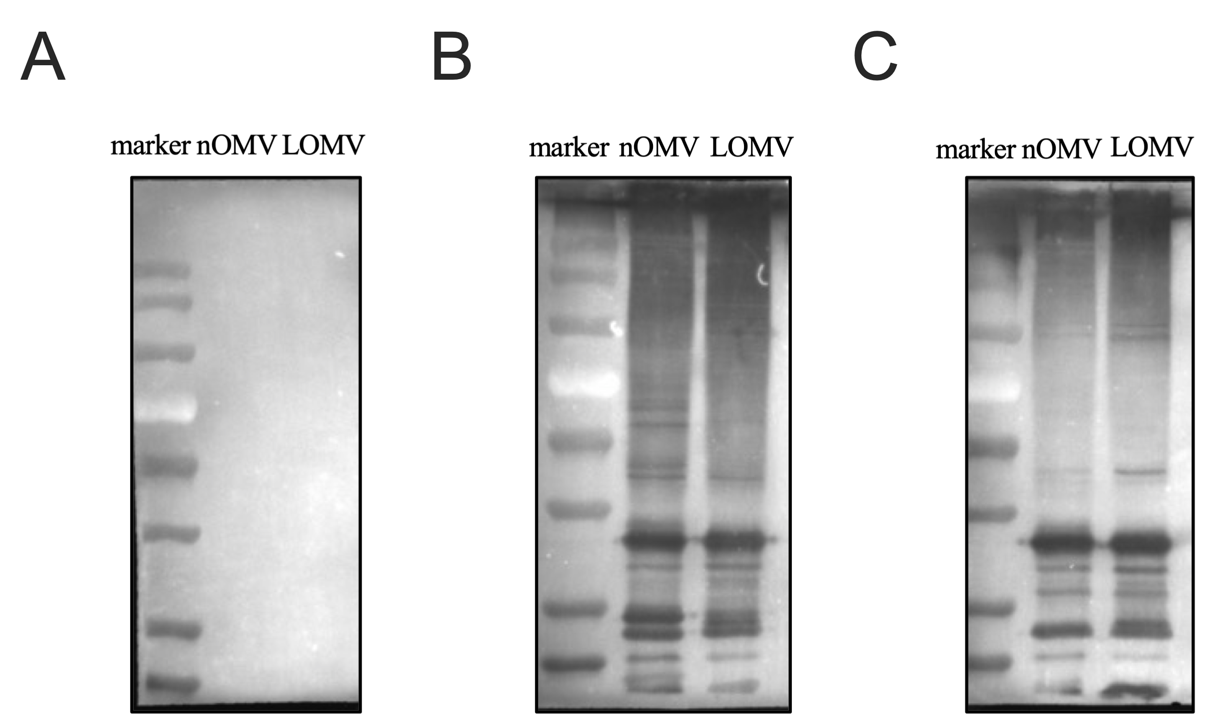


**Figure S4.** Western blots using 42-day serum from intramuscularly vaccinated mice were used to probe OMVs. (A) PBS serum, (B) nOMV serum, (C) LOMV serum.


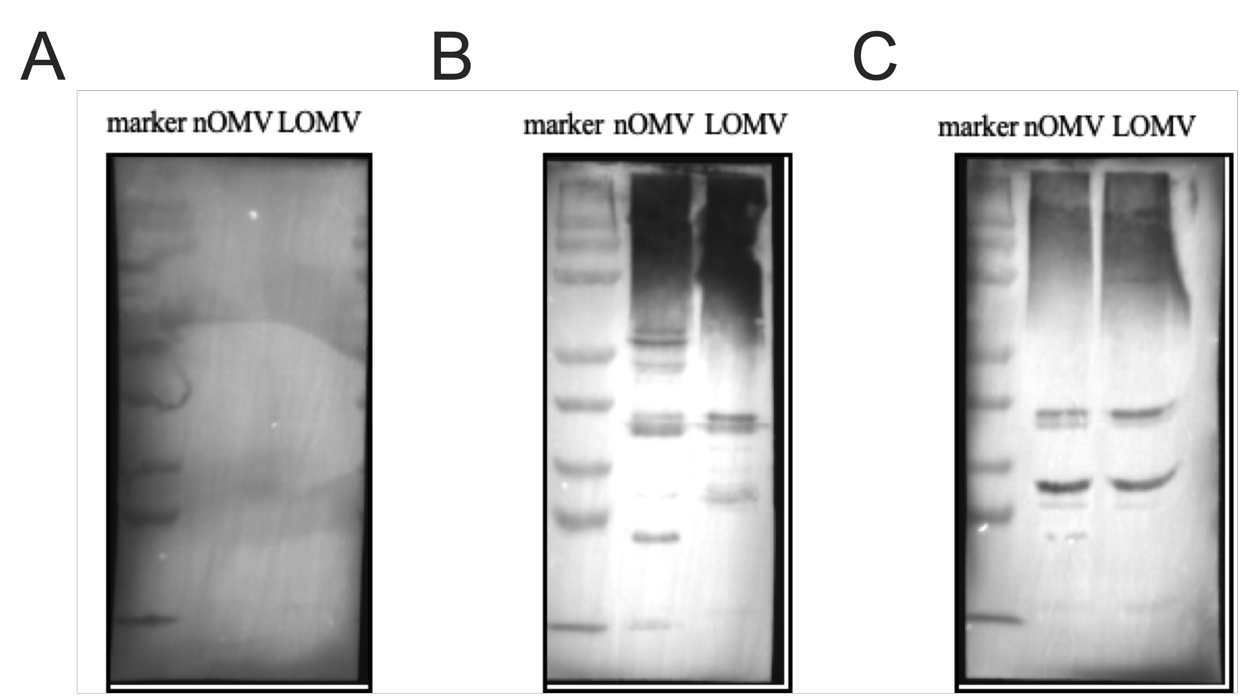


**Figure S5.** Western blots using 42-day serum from intranasally vaccinated mice were used to probe OMVs. (A) PBS serum, (B) nOMV serum, (C) LOMV serum.


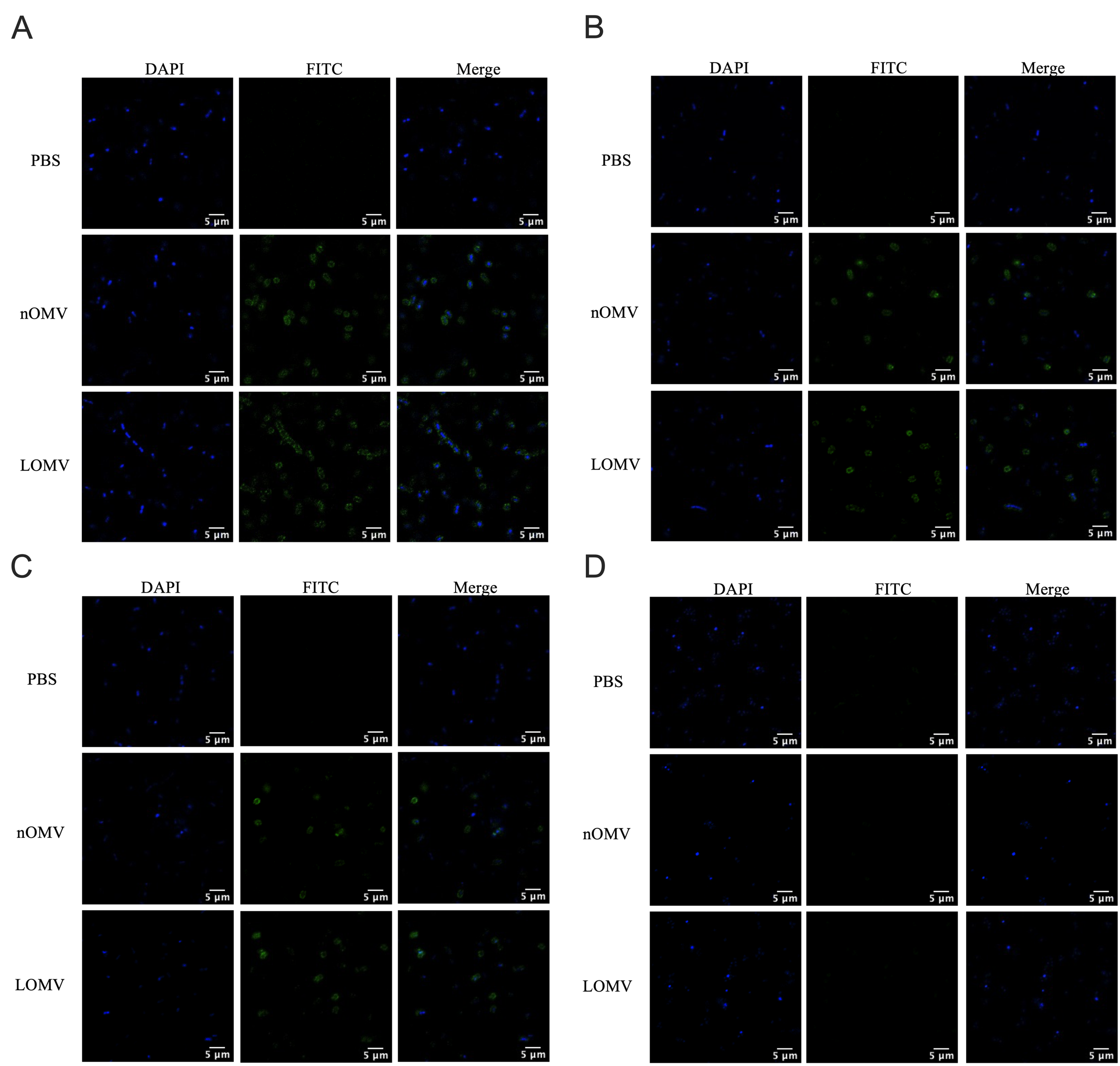


**Figure S6.** Fluorescence confocal microscopy was used to observe the combination of OMVs antibody (intramuscular immunization) and bacteria. (A) *A. baumannii* WHG40137, (B) *A. baumannii* 3, (C) *A. baumannii* LB-6, and (D) *S. aureus* ATCC 29213.


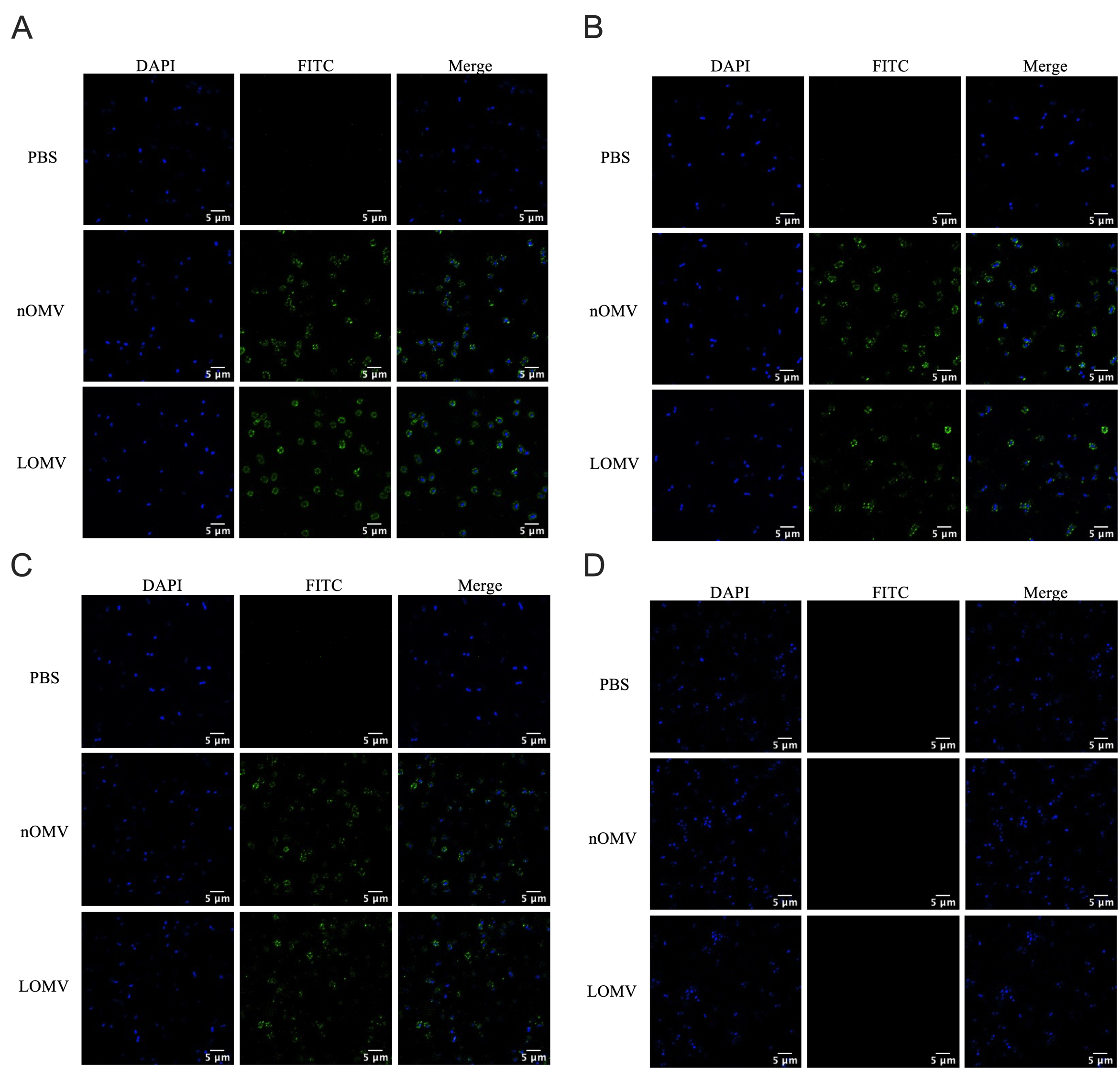


**Figure S7.** Fluorescence confocal microscopy was used to observe the combination of OMVs antibody (intranasal immunization) and bacteria. (A) *A. baumannii* WHG40137, (B) *A. baumannii* 3, (C) *A. baumannii* LB-6, and (D) *S. aureus* ATCC 29213.
